# Comparative Adsorptive Removal of Phosphate and Nitrate from Wastewater Using Biochar-MgAl LDH Nanocomposites: Coexisting Anions Effect and Mechanistic Studies

**DOI:** 10.3390/nano10020336

**Published:** 2020-02-16

**Authors:** Omar Alagha, Mohammad Saood Manzar, Mukarram Zubair, Ismail Anil, Nuhu Dalhat Mu’azu, Aleem Qureshi

**Affiliations:** Environmental Engineering Department, College of Engineering A13, Imam Abdulrahman Bin Faisal University, Dammam 34212, Saudi Arabia; msmanzar@iau.edu.sa (M.S.M.); mzzubair@iau.edu.sa (M.Z.); ianil@iau.edu.sa (I.A.); aqureshi@iau.edu.sa (A.Q.)

**Keywords:** date palm derived biochar, MgAl layered double hydroxides, phosphate and nitrate removal, wastewater, removal mechanism

## Abstract

In this study, date-palm biochar MgAl-augmented double-layered hydroxide (biochar–MgAl–LDH) nanocomposite was synthesized, characterized, and used for enhancing the removal of phosphate and nitrate pollutants from wastewater. The biochar–MgAl–LDH had higher selectivity and adsorption affinity towards phosphate compared to nitrate. The adsorption kinetics of both anions were better explained by the pseudo-first-order model with a faster removal rate to attain equilibrium in a shorter time, especially at lower initial phosphate-nitrate concentration. The maximum monolayer adsorption capacities of phosphate and nitrate by the non-linear Langmuir model were 177.97 mg/g and 28.06 mg/g, respectively. The coexistence of anions (Cl^−^, SO_4_^2−^, NO_3_^−^, CO_3_^2−^ and HCO_3_^−^) negligibly affected the removal of phosphate due to its stronger bond on the nano-composites, while the presence of Cl^−^ and PO_4_^3−^ reduced the nitrate removal attributed to the ions’ participation in the active adsorption sites on the surface of biochar–MgAl–LDH. The excellent adsorptive performance is the main synergetic influence of the MgAl–LDH incorporation into the biochar. The regeneration tests confirmed that the biochar–MgAl composite can be restored effortlessly and has the prospective to be reused after several subsequent adsorption-desorption cycles. The biochar-LDH further demonstrated capabilities for higher removal of phosphate and nitrate from real wastewater.

## 1. Introduction

Nitrate and phosphate are necessary nutrients for the growth of plants, wildlife, and humans. However, elevated concentrations of nitrate and phosphate in surface and groundwater is a severe global concern leading to devastating impacts on ecosystems [1,2]. General sources of nitrate and phosphate contaminants in water bodies usually arise from the waste products of human activities such as discharges from industrialized practices, agricultural uses like inorganic fertilizers, compost, and wastewater treatment effluents [3]. A phosphate generally arises from the element named phosphorous, and affects water quality by the disproportionate development of algae. Phosphates ions present in the water nourish algae, which further destroy other forms of life and yield unsafe contaminants. Excessive concentration of phosphorous results in the eutrophication process, which decreases the amount of dissolved oxygen in the aquatic streams of watercourses and ponds [4]. Likewise, excessive levels of nitrates are considered the main harmful waste for ground and surface waters, and pose a severe threat to the survival of aquatic life. According to the World Health Organization, the acceptable limits of nitrate and phosphate ions in drinking water are 40 mg/L and nearly 0.1 mg/L, respectively [5]. Therefore, there is an urgent need to propose sustainable and cost-effective engineering technologies to remove excess phosphate and nitrate ions in wastewater streams effectively before discharging them to the receiving bodies or their reuse.

Various physicochemical techniques, including chemical precipitation [6], a membrane process [7], oxidation [8], electrodialysis [9,10], constructed wetlands [11], and adsorption [12,13] have been employed as the route to eliminate nitrates and phosphates from aqueous systems. Among all of them, adsorption proved to be one of the most popular techniques because of its low cost, ease of operation, higher removal efficiency, and excellent reusability performance. In the adsorption process, the selection of proper adsorbent material is the key challenge; therefore, researchers and scientists are exerting enormous efforts to produce sustainable, economical, and less toxic adsorbents for the improved remediation performance of contaminants from water streams [14,15].

Biochar is a carbon-rich product obtained from pyrolysis of biomass that has attracted remarkable interest for application in soil improvement and removal of toxic pollutants from the aqueous medium. The porous surface morphology of biochar is highly favorable for holding water and water-soluble nutrients [16]. Previous studies demonstrated biochar as a promising, eco-effective adsorbent due to its excellent physical characteristics, abundant surface functionalities, and superb ion-exchange capability [17,18]. The porous structure with a relatively high specific surface area of the different produced biochars showed enhanced uptake of various toxins [19].

Xiaoning Liu et al. [20] investigated calcium-activated biochar for the remediation of phosphate ions from phosphorus-rich waste streams, and revealed excellent removal efficiency with maximum sorption capacity of 197 mg/g. Ren Jing et al. [21] demonstrated biochar derived from the cotton stalk as an effective phosphate removal adsorbent from water. Chemically modified biochar [22] showed improved removal of nitrate from water solution and 28.21 mg/g of adsorption capacity.

Layered double hydroxides (LDH) are classified as a clay consisting of metal hydroxides layers sandwiched with anions. These materials are represented generally as [M^2+^_(1−*x*)_M^3+^*_x_*(OH)_2_]^x+^ [A^n−^]*_x_*_/*n*_·*m*H_2_O, where M^2+^ and M^3+^ represent the double- and triple-charged positive metal ions, A*^n^*^−^ is the intermediate negative ions, respectively. The versatile composites with outstanding surface and structure characteristics and excellent ion exchange capabilities attracted considerable attention for catalysis [23], purification [24], optics [25], and energy applications [26]. Ronghua Li et al. [27] developed Mg/Al-layered double hydroxide at different mole ratios and revealed improved phosphate adsorption with increasing Mg/Al ratio in the Mg/Al–LDHs biochar composites capacity. Yuki Kamimoto et al. [28] concluded excellent adsorption performance of nitrate onto magnetic calcined Mg–Fe LDH. Muayad Al Jaberi et al. [29] prepared CaFe-layered double hydroxide by co-simple co-precipitation technique and reported a maximum removal capacity of 130 mg/g.

In recent years, substantial research has directed interest in developing biochar-based derivatives as super adsorbents for enhanced remediation of various toxic contaminants from an aqueous medium [30,31]. In particular, coupling biochar with LDH showed a promising and sustainable approach to significantly improve adsorbent characteristics and removal efficiency of pollutants due to the synergetic effect of biochar, and LDHs led to an improvement in surface active sites and thus resulted in enhanced sorption performance. 

In 2019, Qing Liang Cui et al. 2019 [32] investigated magnetic biochar/Mg–Al layered double hydroxide composite and reported relatively higher adsorption capacity as compared to other magnetic adsorbents, with moderate 51.43% removal efficiency. Yan-Hong Jiang et al. [33] loaded ZnAl–LDH on to modified banana straw biochar via a hydrothermal process and demonstrated significant improvement in phosphorus removal. These studies indicated that the intercalation of LDHs into biochar could significantly improve the uptake of phosphate. Therefore, it is expected that the incorporation of MgAl LDHs into date palm-derived biochar could be a sustainable and promising approach to improve the adsorption performance of date palm derived biochar. In addition, the aforementioned studies produced a biochar-LDHs composite via a hydrothermal technique and investigated the removal of phosphate ions only. Therefore, this study investigated the production of biochar derived from date palm from waste intercalated with a MgAl-layered double hydroxides composite via simple co-precipitation technique and explored it for the recovery of both phosphate and nitrate from wastewater streams.

The main objective of this work is to enhance phosphate and nitrate recovery from the water phase using date palm waste-derived biochar augmented with MgAl layered double hydroxides. The synthesized nanocomposites characterization using Fourier transform infrared spectroscopy (FTIR), scanning electron microscopy (SEM), X-ray diffraction (XRD), and Brunauer–Emmett–Teller (BET) measurement techniques to examine the surface and structure properties after the inclusion of LDH layers into biochar. The study focused on the effects of several influencing parameters such as contact time, adsorbent dosage, pH, anions concentration, and temperature on the percentage removal of phosphate and nitrate ions. The adsorption capacity and interaction mechanism of the synthesized composite with phosphate and nitrate anions were evaluated in depth by considering isotherms, kinetics, and thermodynamic models. 

## 2. Materials and Methods 

### 2.1. Chemicals

The production of the biochars used in this study was done at slow pyrolysis (700 °C and four h at a heating rate of 3 °C/min) using date palm waste fronds collected from Al-Hasa city, Saudi Arabia. The pure salts of aluminum (III) nitrate nonahydrate and magnesium (II) nitrate hexahydrate, were obtained from Sigma Aldrich (Darmstadt, Germany) and used without any pretreatment. Potassium dihydrogen phosphate (KH_2_PO_4_), sodium nitrate (NaNO_3_), and potassium sulfate (K_2_SO_4_) were from Sigma Aldrich (Darmstadt, Germany). Hydrochloric acid (HCl) and sodium hydroxide (NaOH) were from Merck (Darmstadt, Germany). The salts of KH_2_PO_4_ and NaNO_3_ were the sources of phosphate and nitrate solution used in the study. Preparation of a stock solution of 1000 mg/L for the experimental work used deionized water. Phosphate and nitrate concentrations were measured using an ultraviolet–visible (UV–visible) spectrophotometer (DR 6000, Hach Lange, Mena, Dubai, UAE). The initial desired value of pH was adjusted using HCl and NaOH.

### 2.2. Synthesis of Date Palm-Derived Biochar-Decorated MgAl Composites

The production of biochar-modified MgAl-layered double hydroxide composites were from different masses of biochar (5 and 10 g) with a 3:1 mole ratio of magnesium (7.69 g) and aluminum (3.75 g) nitrate salts (M^2+^:M^3+^) via co-precipitation method. In detail, first, we dissolved a measured amount of salts in a reaction vessel containing 100 mL of double-distilled water. Simultaneously, a precisely known quantity of biochar derived date palm fronds (5 and 10 g) was also added in 100 mL deionized water, sonicated at 60 amplitude for 30–60 minutes, respectively and transmitted to the reaction flask. The mixture of salts and biochar was stirred vigorously at 800–1000 rpm for 10 min at 90 °C. Afterward, the pH of the reaction mixture was maintained at 10 ± 0.5 by adding 1 M of NaOH dropwise. When the required pH value was achieved, the reactor was kept for nearly 24 h reflux at 90 °C. After the reaction, the resultant precipitates were centrifuged and washed 3–4 times with double-distilled water, and finally by ethanol to remove all unreacted salts and impurities. The final product biochar decorated MgAl composite was kept at 60 °C in the oven for 48 h. The resultant composites were then stored in a desiccator for future experiments. The actual compositions of biochar–MgAl composites are listed in Table 1.

### 2.3. Characterization of Biochar-Decorated MgAl Composites

The biochar decorated MgAl composites characterized by Fourier transform infrared spectroscopy (FTIR, Nicolet 6700, Thermo Fisher Scientific, Waltham, MA, USA, resolution 4 cm^−1^), X-ray diffraction (XRD, D8 advance X-ray instrument, Waltham, MA, USA, wavelength = 0.1542 nm, and 2θ = 10° to 80°), scanning electron microscopy (SEM, SM-6460LV (Jeol), Peabody, MA, USA), transmission electron microscopy (FEI, Morgagni 268, Berno, Czech Republic), and Brunauer–Emmett–Teller measurement (BET Tristar II series, Micromeritics, Norcross, GA, USA).

### 2.4. Adsorption of Phosphate and Nitrate onto Biochar MgAl Composites

A preliminary adsorption experiment was performed using biochar and biochar-decorated MgAl composites to evaluate the removal performance of nitrate and phosphate and select the adsorbent showing better adsorptive performance for detailed adsorption studies. Batch mode adsorption experiments were performed on the adsorbent chosen to investigate the effect of adsorption parameters including initial solution pH 2–12, adsorbent amounts of 5–100 mg, anion concentration between 10–50 mg/L, contact time within 10–840 min, and temperature (298–318 K) in temperature-controlled agitator at fixed rpm of 275 containing 40 mL of solution in 50 mL plastic vials. All the experiments were performed in duplicate and the average values reported herein. 

The removal efficiencies and adsorption capacities were determined by using the following Equations (1) and (2):

(1) $$\mathrm{Removal}\;\mathrm{efficiency}\;\left(\%\right)=\frac{C_{o}-C_{e}}{C_{o}}\times 100$$

(2) $$q_{e}=\left(C_{0}-C_{e}\right)\times \frac{V}{m}$$

where *C_o_* and *C_e_* are the initial and the equilibrium concentrations (mg/L) of PO_4_^3−^ and NO_3_^−^, respectively, *q_e_* is the equilibrium amount of PO_4_^3−^ and NO_3_^−^ adsorbed (mg) per unit mass of adsorbent (mg/g), *V* is the volume of the solution (L), and m is the mass of the adsorbent in the solution (g).

### 2.5. Co-Existing Ions Experiment

There are a lot of anions present in the water or groundwater such as carbonate, bicarbonate, nitrate, sulfates, chlorides, and phosphates, etc. which might prevent the removal of nitrate or phosphate during a sorption process. Therefore, the influence of these anions (Na_2_CO_3_, NaCl, Na_2_SO_4_, NaHCO_3_, and NaNO_3_) was investigated at varied concentration from 0.001 to 0.1 M. The co-existing anions sorption experiment was conducted by using 40 mL of reaction mixture containing specified concentration of co-existing anions and 10 mg/L of phosphate and nitrate ions agitated with 5 mg of B_5_MgAl composite at 200 rpm for 24 h.

### 2.6. Adsorption Isotherm and Kinetic Modeling

The isotherm and kinetic experiments were performed at various phosphate and nitrate concentrations ranging between 10–50 mg/L, contact time between 10–840 minutes, and variable temperature values (298–318 K) at constant parameters including composite dosage of 5 mg, pH 6, and agitation speed of 275 rpm. The final concentrations of the samples were estimated using a barcoded test kits on the UV–visible spectrophotometer. The adsorption mechanism of phosphate and nitrate onto biochar MgAl composite was demonstrated by utilizing kinetics, isotherm models as well as characterization of the spent composites. Thus, in this study, three kinetic models (pseudo-first, second-order) were used while the linear and non-linear form of Langmuir and Freundlich’s models were employed for the equilibrium study. These models equations and the respective estimated models’ parameters are listed in Section 3.5 “Adsorption Rate and Kinetic Modeling” and Section 3.6 “Isotherm Modeling”, respectively.

### 2.7. Sequential Adsorption-Regeneration Cycles 

The adsorption-regeneration experiment was conducted by shaking 200 mL of known concentration of phosphate and nitrate (10.0 mg/L) with 50 mg B_5_MgAl composite at 200 rpm and 24 h. After the adsorption experiment, the supernatant was separated from the spent sorbent with the help of filtration followed by centrifugation (5000 rpm and 5 min). The final concentration of the supernatant was measured, as indicated in Section 2.1. The spent B_5_MgAl composite was regenerated with two different levels of NaOH (0.1 and 1.0 M). The recyclability of the B_5_MgAl composites was assessed by accomplishing the five consecutive adsorption-regeneration sequences. The successive adsorption/regeneration experiments repeated, and the average values indicated in results.

## 3. Results and Discussion

### 3.1. Characterization of Biochar–MgAl Composites

**Figure 1:**
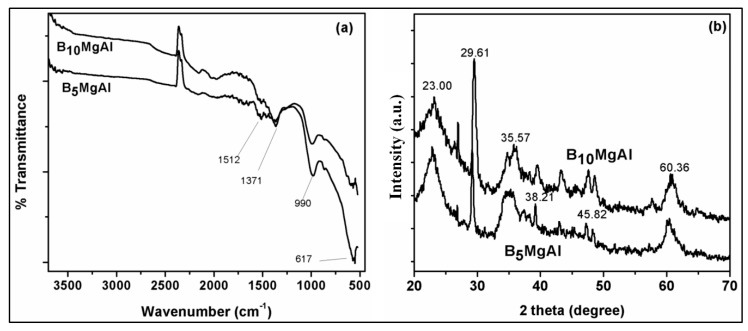
Fourier transform infrared (FTIR) spectra of biochar/MgAl composites (**a**), X-ray diffraction (XRD) pattern of biochar–MgAl composites (**b**).

Figure 1a,b displays the FTIR and XRD spectrums of B_5_MgAl and B_10_MgAl composites, respectively. The FTIR spectrum of both composites showed the presence of surface functionalities associated with biochar and MgAl LDH. For instance, the sharp broadband at 1371 cm^−1^ was attributed to the stretching vibration of nitrate in the interlayer space of the LDH [34]. Similarly, the characteristic peaks of C–O and mixed metal oxides (MM–O) were observed at 980 and 870 cm^−1^, respectively. The sharp peak at 617 cm^−1^ was ascribed to lattice vibrations of the MM–O bonds [35]. Also, the crystallographic structure results of the biochar–MgAl composites assessed by XRD patterns are shown in Figure 1b. The sharp and broad diffraction peaks located at 2θ = 23.00°, 29.61° and 60.36° correspond to quartz (SiO_2_), potassium aluminate (KAlO_2_), and dolomite (CaMg(CO₃)₂), respectively, indicating excellent intercalation of biochar into LDH layers without damaging the crystalline structure of the composite with slightly deviation at increased biochar content. The results clearly demonstrated that the synthesized composite is functionalized with different oxygen functionalities (OH, C–O–C, NO_3_^−^, MM–O) and excellent crystallinity which may expected to provide improved uptake of phosphate and nitrate from water phase.

**Figure 2:**
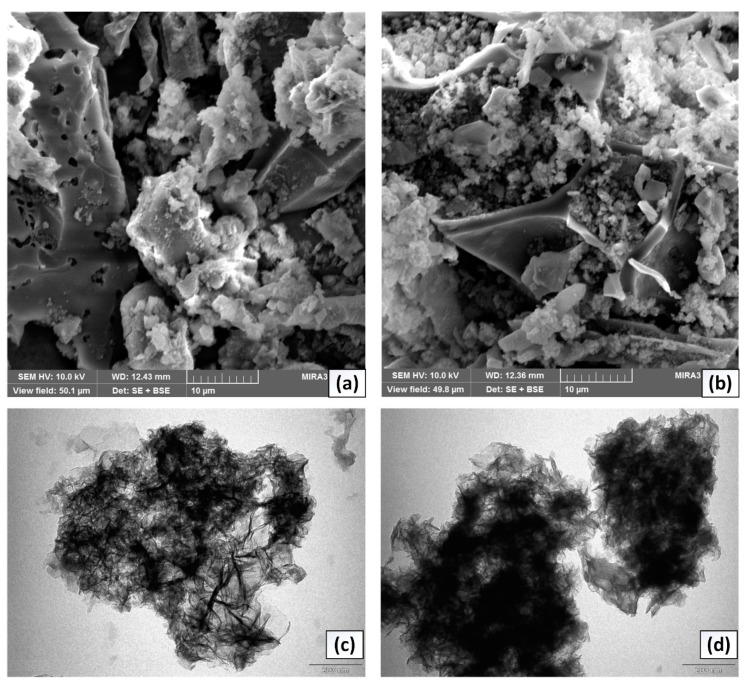
SEM images of (a): B_5_MgAl and (b) B_10_MgAl composites and TEM images of (c) B_5_MgAl and (d) B_10_MgAl composites.

The dispersion of biochar into layers of MgAl LDH further demonstrated by analyzing surface morphology of composites using SEM and transmission electron microscopy (TEM, Morgagni 268, FEI Company, Brno, Czech Republic) techniques (Figure 2). The SEM images indicated rough heterogeneous structure comprised of tiny particles surrounded in the entire surface. Moreover, as the biochar amount increased from 5 to 10 g, the proportion of small particles associated with biochar increased, leading to smooth and homogenous surface morphology. TEM analysis carried out to explain the dispersion of biochar at various mass ratios indicates that in B_5_MgAl composite, the particles of biochar homogeneously intercalated throughout the layers of MgAl LDH, which suggests substantial enhancement in surface and structure properties and favorable uptake of contaminants. Moreover, at a higher biochar amount (see B_10_MgAl), biochar particles seem to agglomerate within the layers of LDH reflecting breakage of the LDH structure and this is expected to reduce the anion removal efficiency.

**Figure 3:**
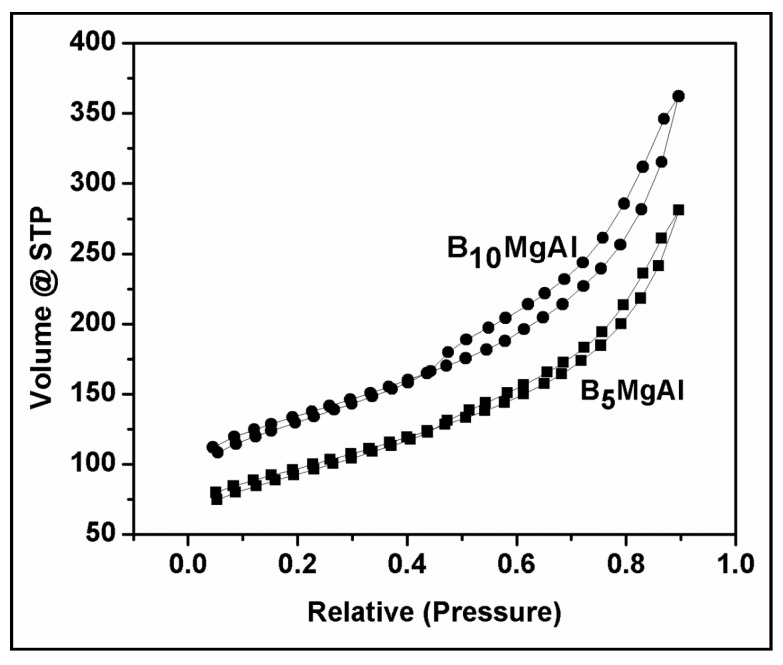
N_2_ adsorption/desorption of biochar/MgAl composites.

The N_2_ adsorption-desorption isotherm (Figure 3) used to calculate the specific surface area, average pore radius, and volume of B_5_MgAl and B_10_MgAl composites and their values are listed in Table 2. Results show that the B_5_MgAl composite exhibited better textural characteristics (surface area and pore volume) compared to B_10_MgAl and other reported biochar and LDH composites. Improved surface area and pore volume of B_5_MgAl indicated better interaction of biochar with MgAl surface whereas lower textural properties of the high loading of biochar (B_10_MgAl) could lead to agglomeration of biochar within the layers of MgAl LDH (as confirmed from TEM results) and this is expected to reduce the sorption performance [36].

### 3.2. Phosphate and Nitrate Adsorption Performance of Biochar and Biochar–MgAl Nanocomposite

**Figure 4:**
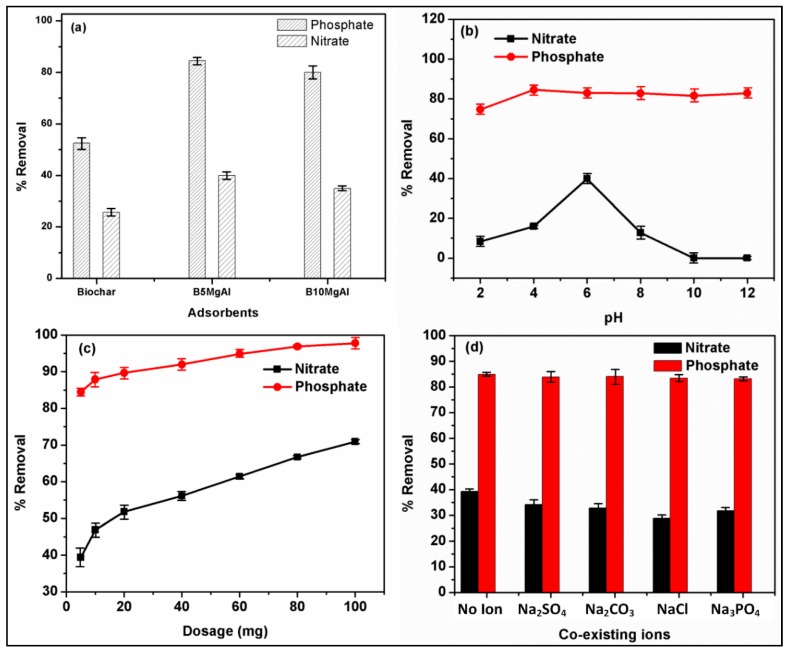
Preliminary experiment of adsorption of phosphate and nitrate by biochar and biochar/MgAl composite (**a**), effect of initial pH (**b**), dosage (**c**), and co-existing ions 0.001 M (**d**) on the percentage removal of nitrate and phosphate onto biochar/MgAl composite.

Results of the preliminary batch-test performed for the percentage removal performance of biochar and biochar-decorated MgAl composites (B_10_MgAl and B_5_MgAl) are displayed in Figure 4a. It was found that the percent removal of virgin biochar significantly increased by incorporating with MgAl LDH. B_5_MgAl composite yielded better performance for phosphate and nitrate removal compared to the B_10_MgAl composite. The high performance was attributed to better intercalation of biochar into MgAl LDHs interlayers leading to significant improvement in surface functionalities, improved textural properties, and heterogeneous surface morphology with excellent crystallinity as confirmed by the characterization results (Section 3.1). Therefore, B_5_MgAl composite was selected for further adsorption experiments whose results are discussed below.

### 3.3. Influential of Adsorption Parameters on Percentage Removal of Nitrate and Phosphate

The effect of pH on the removal of nitrate and phosphate onto B_5_MgAl composite is displayed in the (Figure 4b). As shown, the removal efficiency of phosphate increased as the initial pH increased from 2 to 4. Further increase in pH value from 4 to 12 showed almost minimal change in percent removal of phosphate. The highest removal of phosphate was observed between pH 4 to 6. Similarly, in the case of nitrate, there was a substantial improvement in the removal efficacy (10% to 40%) when the pH was increased from 2 to 6, respectively. However, a significant reduction was observed with further change in pH above 6. To further illustrate the pH effect on removal performance, point of zero charges (pH_PZC_) of B_5_MgAl were estimated using a pH drift method, and its value found as 10.02. Therefore, the surface charge of B_5_MgAl was entirely protonated at pH < pH_PZC_ = 10.02. In acidic medium, phosphate mainly exists in two forms individually as HPO_4_^2−^ and H_2_PO_4_^−^, thus at weakly acidic conditions (4 < pH < 6), the positively charged surface of B_5_MgAl strongly interacted with negative phosphate anions via electrostatic attractions.

On the other hand, at solution pH > pH_PZC_, the surface will become deprotonated, causing electrostatic repulsion with negatively charged anions, resulting in a decrease in percentage removal. The results further indicated that the synthesized biochar–MgAl–LDH composite is highly efficient for the removal of phosphate and nitrate at pH ranges (4–10) and (neutral), respectively. Therefore, based on pH results and considering the chemical stability of LDH due to dissolution at powerful acidic medium, pH values of 4 and 6 were selected for further phosphate and nitrate adsorption experiments. 

To evaluate the effect of adsorbent dose as it is a known vital factor in the adsorption of pollutants from the aqueous phase, adsorption experiments were conducted at varying B_5_MgAl composite dosage between 5–100 mg at pH 6, initial concentration of 10 mg/L, and temperature of 298 K (Figure 4c). As can be observed, an increase in dosage of B_5_MgAl from 5 mg to 100 mg significantly improved the nitrate removal from 39.4% to 70.9%. Besides, compared to nitrate, B_5_MgAl showed strong interaction with phosphate with almost 84.5% removal at only 5 mg. The higher removal efficiency of phosphate at low B_5_MgAl amount was attributed to the synergetic effect of biochar, and MgAl–LDH was leading to stronger interaction of phosphate molecules with surface functionalities of composite via electrostatic and ion-exchange mechanisms during the adsorption process. 

### 3.4. Effect of Co-Existing Ions

The anions co-existing with phosphate and nitrate in the water solution affect the removal of phosphate and nitrate. The influence of coexisting anions (Cl^−^, SO_4_^2−^, NO^3−^, CO_3_^2−^ and HCO_3_^−^) at varying concentrations (0.001 to 0.1 M) on phosphate and nitrate removal on biochar–MgAl were inspected at phosphate and nitrate solution (10 mg/L), respectively, and are displayed in Figure 4d and Figure S1. A negligible effect of coexisting anions (almost 2% reduction) on phosphate removal by biochar–MgAl was found due to the stronger bonds of Cl^−^, SO_4_^2−^, NO_3_^−^, CO_3_^2−^ and HCO_3_^−^ to surface sites of biochar–MgAl when phosphate and other anions coexisted in the water solution. It was noted that SO_4_^2−^ and CO_3_^2−^ concentrations (0.001–0.1 M) did not appreciably affect phosphate adsorption (Figure 4d and Figure S1). This phenomenon could further be illustrated by the strong bonding between the phosphate and biochar–MgAl surface. Conversely, the nitrate removal reduced to approximately 20–25% and 30–32% in the presence of Cl^−^ and PO_4_^3−^ (at 0.001–0.1 M), respectively, which could be attributed to these ions participated in the active adsorption sites on the surface of biochar–MgAl with nitrate ions. The composite had an excellent selectivity and adsorption affinity towards SO_4_^2−^ and CO_3_^2−^ as compared to Cl^−^ and PO_4_^3−^ ions. The outcome recommended that the given adsorbent had a high adsorption discernment towards phosphate ions as compared to nitrate ions present in the water solution. Moreover, at the presence of Na_3_PO_4_ and NaCl, the percentage removal of nitrates dropped from 31.8% to 30.2% and from 28.9% to 28.4%, respectively. There was a minimal decrease in the percentage removal of nitrates at a lower level (0.001) M of Na_2_SO_4_ and Na_2_CO_3_ (Figure 4d). However, high concentration (0.1 M) of Na_2_SO_4_ and Na_2_CO_3_ resulted in substantial decrease (25–30%) in percent removal of nitrate (Figure S1). In fundamental solutions, the higher levels of OH^−^ ions would be a hurdle to phosphate and nitrate adsorption as clearly shown (Figure 4d). Consequently, a solution with high pH was unfavorable to the phosphate and nitrate adsorption on the biochar–MgAl. The percentage removal of phosphate by biochar–MgAl was superior to that of nitrate in the presence of SO_4_^2−^, CO_3_^2−^, Cl^−^, and PO_4_^3−^ ions. The results agree with previously reported studies on anions like Cl^−^, SO_4_^2−^, NO_3_^−^, CO_3_^2−^ and HCO_3_^−^ affecting nitrate or phosphate removal.

### 3.5. Adsorption Rate and Kinetic Modeling

**Figure 5:**
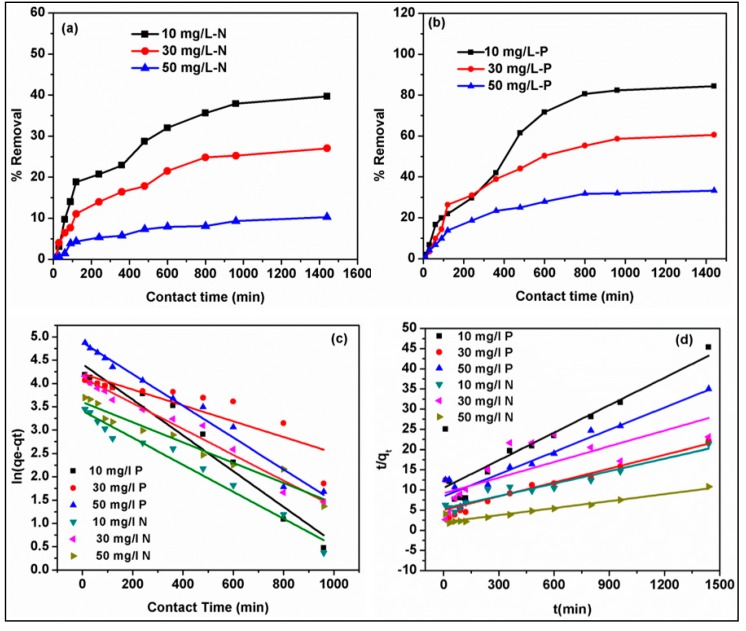
Rate of adsorption of nitrate and phosphate (**a**,**b**), linear plots of pseudo-first-order and pseudo-second-order kinetic models (**c**,**d**) for the removal of nitrate and phosphate onto biochar–MgAl composites.

The evaluation of the adsorption rate of phosphate and nitrate onto B_5_MgAl composite was conducted at three different concentrations (10, 30, and 50 mg/L) with different contact times (0–840 min) while other parameters such as pH, temperature, dosage were kept constant. As shown in Figure 5a,b, for both nitrate and phosphate, the rate of adsorption was fast at the first 120 min followed by slow adsorption and reached equilibrium within 180–600 minutes for 10–50 mg/L of anions concentrations. The rapid adsorption rate was attributed to the availability of active binding sites on the B_5_MgAl surface leading to quick uptake and intense interaction with anion molecules, which is comparatively faster than previously reported biochars and their derivatives for phosphate and nitrate removal. 

To further examine the sorption behavior of nitrate and phosphate, pseudo-first-order, and pseudo-second-order models were applied to the experimental data. The significant factors and correlation coefficients (*R*^2^) of kinetics models are listed in the above Table 3 and their respective plots depicted in Figure 5c,d. The regression coefficients *R*^2^ of the pseudo-first-order model varied between 0.951–0.988 in 10 mg L^−1^ of nitrate and phosphate ions at 303 K, which is higher than pseudo-second-order model *R*^2^ ranging from 0.802–0.938 [37]. The high value indicates that the pseudo-first-order model better explained the adsorption process. The adsorption process of B_5_MgAl on the removal of nitrate and phosphate relates to the physic-sorption method between the dynamic sorbent sites and phosphate-nitrate ions. As compared to other studies, the rate constants of the pseudo-first-order model (*k*_1_) were 0.0057 and 0.0108 g/(mg min) at the initial concentrations of 10 mg/L phosphate and nitrate respectively was lower. For example, Xiaolan Hu et al. [38] calculated the rate constants for the first-order model *k*_1_ 0.381 min^−1^.

Similarly, Zhanghong Wang et al. [39] calculated rate constants *k*_1_ for the first-order kinetics model 9.47 min^−1^ that were still higher than the present study. Thus, it is concluded that B_5_MgAl composite holds a quicker removal rate at a lower initial phosphate-nitrate concentration and probably will attain equilibrium in a shorter period. These outcomes demonstrated that a shorter time is required for the B_5_MgAl composite to remove phosphate and nitrate effectively from the aqueous medium. Furthermore, the experimental value *q_e_* is almost similar to the *q_e_* value obtained in the pseudo-first-order kinetic model.

### 3.6. Isotherm Modeling

**Figure 6:**
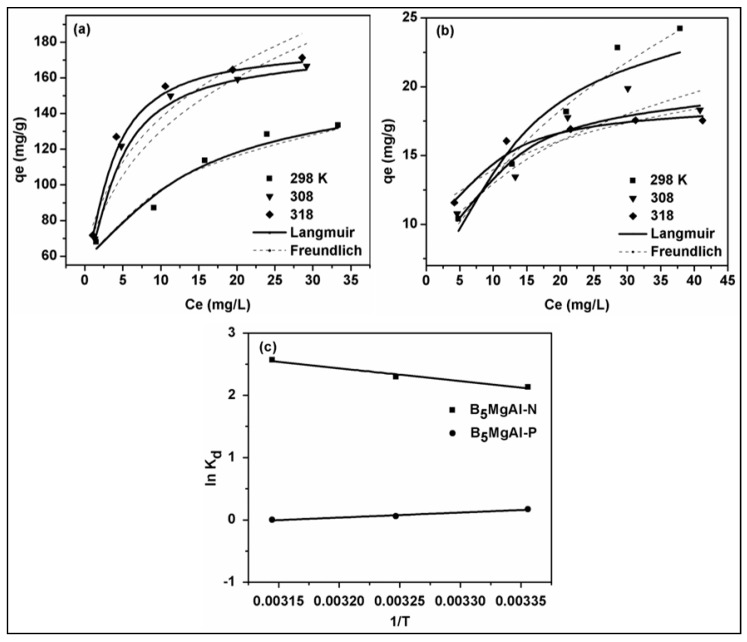
Non-linear isotherm models of phosphate (**a**) and nitrate (**b**) removal onto biochar/MgAl composites, plot of ln*K_d_* against 1/*T* for the estimate of thermodynamic parameters (**c**).

The interaction of adsorbate (phosphate and nitrate) on the surface of B_5_MgAl composite was further investigated via commonly used isotherm models, namely the Langmuir and Freundlich models. The equilibrium data fitted on linear and non-linear models equations at three different temperatures. The calculated parameters from linear and non-linear equations and non-linear fitted curves are displayed in Table 4 and Table 5 and Figure 6, respectively. Considering the linear and non-linear correlation coefficient values, the Langmuir isotherm model (*R*^2^ > 0.982) revealed better fitting than the Freundlich model (*R*^2^ > 0.930) for phosphate adsorption. However, for nitrate, both Langmuir and Freundlich showed better fits with *R*^2^ > 0.986 at all studied temperatures. The estimated maximum adsorption capacities of phosphate and nitrate by the non-linear Langmuir model were 41.75, 176.09, and 177.97 mg/g and 28.06, 20.78 and 19.00 mg/g at 298, 308 and 318 K, respectively. The values of *K_L_* (indicates the level of interaction between sorbate and sorbent) are higher for phosphate than nitrate which suggests that phosphate species more strongly interacted with surface functionalities on to biochar–MgAl composite. The value of 1/*n* in the Freundlich isotherm model is the measure of concentration effect on adsorption performance, ranging from 0 to 1, and this was favorable adsorption. In our study, the 1/*n* value of B_5_MgAl for phosphate adsorption was between 0.22 and 0.28 demonstrating better adsorption than that of nitrate (1/*n* = 0.183 − 0.425). The excellent adsorption behavior of as B_5_MgAl confirmed that the coupling of MgAl with date palm-derived biochar via the co-precipitation technique is a promising approach to produce adsorbent for the recovery of phosphate and nitrate from wastewater streams. Also, the improved adsorption capacity at elevated temperature suggests that higher temperature is favorable for phosphate removal onto B_5_MgAl resulting in better interaction forces between adsorbent and phosphate anions. The enhanced adsorption further confirms that chemical interactions were mainly associated with the sorption of phosphate. Consequently, this corroborates results reported by Hui He et al. [40] that investigated MgAl-decorated tobacco-stalk biochar for phosphate recovery. In contrast, nitrate adsorption performance showed a reduction in removal efficiency with increased adsorption temperatures. The previous reduction demonstrates the exothermic nature and physisorption mechanism of nitrate onto B_5_MgAl composite.

### 3.7. Thermodynamic Modeling

During the adsorption process, changes in the thermodynamic parameters including standard free Gibbs energy (Δ*G*), the standard enthalpy change (Δ*H*), and the standard entropy change (Δ*S*) control the mechanism and feasibility of the adsorption. The Van’t Hoff equations in Equations (3) and (4) were used to calculate these thermodynamic parameters [41,42].

(3) $$\ln\left(K_{d}\right)=-\frac{\Delta H}{R}\frac{1}{T}+\frac{\Delta S}{R}$$

(4) ΔG=ΔH−TΔS

where *K_d_* is the thermodynamic equilibrium constant calculated by plotting ln(*q_e_*/*C_e_*) against *q_e_* where *q_e_* extrapolated to zero [34]; R is the universal gas constant (8.314 J/mol K); *T* is the absolute temperature (K). The thermodynamic parameters Δ*H* and Δ*S* are calculated by plotting ln*K_d_* against 1/*T*.

The thermodynamic parameters, including *K_d_*, Δ*H*, and Δ*S*, and Δ*G*, computed for both phosphate and nitrate adsorption at three different temperatures of 298, 308, and 318 K are given in Table 6. Accordingly, the thermodynamic parameters were assessed to reveal the effect of temperature on phosphate and nitrate adsorption onto B_5_MgAl composite. The negative Δ*G* values found for both pollutants suggest spontaneous adsorption processes. Values of Δ*G* ranging between 0 and −20 kJ/mol ascribed to physical adsorption [43,44]. Accordingly, the phosphate adsorption processes on B_5_MgAl composite was endothermic due to the positive value of Δ*H*, whereas negative Δ*H* for nitrate indicates an exothermic reaction. The Δ*S* values specify the increase in the randomness of the solid/liquid interface during the adsorption process, exhibiting that the uptake of phosphate and nitrate onto B_5_MgAl composite were favorable processes. These thermodynamic modeling results are in good agreement with the findings of previously reported studies using various other MgAl LDHs for phosphate and nitrate adsorption [41,45,46].

### 3.8. Interaction Mechanism of Phosphate and Nitrate with Biochar–MgAl Composite

**Figure 7:**
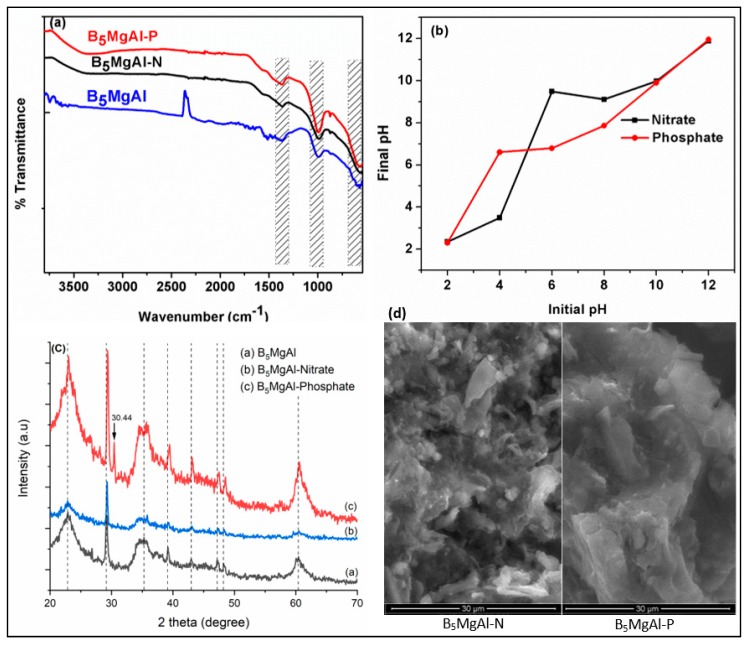
FTIR (**a**), pH after adsorption (**b**), XRD (**c**), and SEM images (**d**) of B_5_MgAl before and after phosphate and nitrate adsorption.

FTIR, XRD, and SEM analyses were performed for B_5_MgAl composite after anions adsorption to provide further insight into possible adsorption mechanisms. The FTIR spectra of studied B_5_MgAl composite before and after phosphate and nitrate adsorption are compared in Figure 7a. The involvement of MM–O bonds in phosphate and nitrate adsorption slightly weakened the strength of MM–O stretching vibrations observed between 540 and 690 cm^−1^. A broad absorption band around 3370 cm^−1^ appears after phosphate and nitrate adsorption, which was attributed to the O–H stretching vibrations of interlayer water molecules [41,47]. The appearance of this band after adsorption is a result of the rehydration of M–M–Os. The intensity of this appeared band after phosphate adsorption was more than that of nitrate adsorption, clarifying the higher adsorption capacity of B_5_MgAl composite toward phosphate than for nitrate. Previous studies reported that the intercalation of phosphate into the interlayer space of the MgAl LDH composite after the adsorption process results in a P–O stretching vibration leading to a distinct band noticed between 1030 and 1100 cm^−1^ [27,40,42,47,48,49,50,51]. Accordingly, a shift of the band from 980 to 1000 cm^−1^ and the relatively stronger right shoulder of the peak at 1000 cm^−1^ after phosphate adsorption is due to the formation of mostly outer-sphere surface complexes and partially inner-sphere surface complexes on the B_5_MgAl composite [47]. 

Figure 7b depicts the variation in equilibrium solution pH after phosphate and nitrate adsorption onto B_5_MgAl composite. The equilibrium solution pH is higher than the initial pH values; 4–6 for phosphate and 6–8 for nitrate, which could be due to the ligand exchange of phosphate and nitrate with OH^−^ on the surface of the B_5_MgAl LDH composite [27,40,48,50,51]. The structure of B_5_MgAl was partially destroyed at pH = 2 and, the released OH^−^ from its structure slightly increased the equilibrium solution pH. The effect of initial pH on phosphate removal efficiency was not significant (< 10%), as indicated in Figure 4b. These results imply that the phosphate adsorption of B_5_MgAl composite is probably due to the formation of outer-sphere and inner-sphere surface complexes on the B_5_MgAl composite. At pH 6, the maximum difference between the initial solution pH and the equilibrium solution pH was accompanied by the highest nitrate adsorption. The nitrate adsorption capacity decreased remarkably against further increases or decreases in pH. The affinity of LDHs for anions follows the order PO_4_^3−^ > OH^−^ > NO_3_^−^ according to the Hofmeister series [46], which could explain the decrease in nitrate adsorption at high pH values.

The XRD patterns of B_5_MgAl composites before and after anions adsorption are given in Figure 7c. The XRD spectrum of B_5_MgAl composite before and after nitrate adsorption indicated a similar crystallographic structure. However, the peak intensities after nitrate adsorption significantly reduced compared to the pre-adsorption B_5_MgAl composite, which is most probably due to the ion exchange resulting from nitrate adsorption [47]. After phosphate adsorption, peak positions slightly shifted to the right, peak intensities significantly increased, and a new peak formed at 30.44° (2θ). Yao*,* et al. [52] reported the formation of new Mg–P crystals in the forms of MgHPO_4_ and Mg(H_2_PO_4_)_2_ (around 30° (2θ)) after phosphate adsorption by Mg–biochar–LDH nanocomposite. Liao, Liu, Li and Yang [46] showed that Mg atoms played a more prominent role in phosphate and nitrate adsorption compared to Al atoms. As a consequence of XRD data assessment, the variations in layer charge density, phosphate intercalation into the interlayer, and formation of inner-sphere complexation contributed to the selectivity and phosphate adsorption capacity of the B_5_MgAl composite. It is worth mentioning that nitrate mostly adsorbed on the surface and the near-edge interlayer but was not intercalated into the interlayer of B_5_MgAl composite.

SEM images of B_5_MgAl composite after anions adsorption shown in Figure 7d. The rough and porous morphology of the pre-adsorption B_5_MgAl composite (see Figure 2) became smooth and non-porous after anions adsorption. The surface of B_5_MgAl composite after phosphate adsorption was softer than that of the composite after nitrate adsorption due to the uniform surface coverage by phosphate ions and higher affinity of the B_5_MgAl composite toward phosphate than for nitrate.

### 3.9. Reusability Performance of Spent Biochar–MgAl Composite

**Figure 8:**
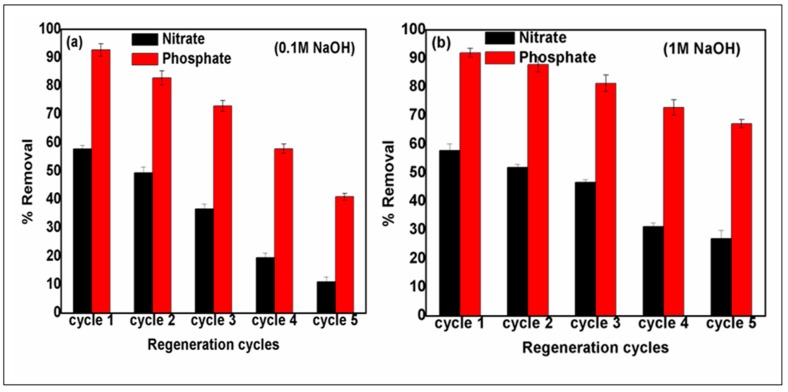
Reusability performance of B5MgAl after regeneration at (**a**) 0.1M NaOH and (**b**) 1M NaOH.

Regeneration was undertaken to find the re-usability of the adsorbent after successive cycles of adsorption-desorption tests. The used B_5_MgAl composite was soaked in the NaOH solutions (0.1 and 1 M) to regenerate it for the next consecutive adsorption-desorption cycles. Typically in basic solutions, the electrostatic interaction between the sorbent and the anionic species weakens because of the removal of H^+^ ions from the solution, and hence desorption of the anions occurs from the adsorbent sites [53]. Figure 8a,b displays the percentage removal of nitrate and phosphate after each regeneration cycle with 0.1 and 1 M NaOH, respectively. As shown in Figure 8a, the adsorption capacities of nitrate and phosphate ions regenerated adsorbents found to be about 20.0 mg/g and 74.5 mg/g respectively even after fifth adsorption-desorption sequence. For the given adsorbent, the percentage removal decreased from 57.9% to 49.5% in the first two sequences and then concluded at 11% in the fifth sequence. Likewise, for phosphate ion, the removal efficiency reduced by about 50% after five sequences (Figure 8a). Moreover, regeneration using 1 M NaOH, resulted in better reusability performance of the biochar–MgAl composite. In detail, the percent removal of nitrate and phosphate (*C_o_* = 10 mg/L) reduced from 57% to 27% and from 92% to 67%, respectively, after five consecutive adsorption-regeneration cycles (Figure 8b). The achieved removal performances of the composite regenerated at 1 M NaOH were higher than those of 0.1 M NaOH 

The regeneration revealed that there was a decline in the percentage removal efficiency after five sequences, indicating that the regeneration cycle was approximately useful for the retrieval of sorption sites on biochar composites. The regeneration test confirmed that the biochar–MgAl composite can be restored effortlessly and has the potential to be recycled after several treatment sequences.

### 3.10. Treatment Performance of Biochar/MgAl for Wastewater Influent

The potential of real application of the new date-palm biochar–MgAl–LDH composite was evaluated for the removal of phosphate and nitrate ions from wastewater influent collected from a wastewater treatment plant (Imam Abdurrahman Bin Faisal University campus). The primary influent sample contained 3.68 and 3.65 mg/L concentration of phosphate and nitrate ions, respectively. The removal of anions by biochar/MgAl (B_5_MgAl) composite were carried out at adsorption conditions (pH 6, dosage 0.125 g/L, contact time 600 min and *T* 25 °C). The percentage removal of phosphate and nitrate in real wastewater by date-palm biochar–MgAl composite obtained 84.80% and 41.90% which is nearly similar to the removal performance for 10 mg/L of anions in synthetic water. These results confirm the synthesized date-palm biochar-decorated MgAl–LDH as a highly promising and sustainable adsorbent material for efficient recovery of phosphate and nitrate ions from real wastewater streams.

## 4. Conclusions

This paper reports the enhanced phosphate and nitrate recovery from the wastewater phase using date palm waste-derived biochar decorated with MgAl-layered double hydroxides. The synthesized composites were characterized by FTIR, SEM, TEM, XRD, and BET techniques to examine the surface and structure properties after the inclusion of LDH layers into biochar. The performance of the new nanocomposite was investigated under several influencing parameters such as contact time, dosage, pH, anions concentration, and temperature. The adsorption capacity and interaction mechanism of the synthesized composite with phosphate and nitrate anions were evaluated in depth by considering isotherms, kinetics, and thermodynamic models. The presence of coexisting anions (Cl^−^, SO_4_^2−^, NO_3_^−^, CO_3_^2−^, and HCO_3_^−^) negligibly affected the removal of phosphate attributed to its stronger bond on the composites, while the presence of Cl^−^ and PO_4_^3−^ reduced the nitrate removal as a result of these ions participating in the active adsorption sites on the surface of biochar–MgAl with nitrate ions. The composite had a superior selectivity and adsorption affinity towards SO_4_^2−^ and CO_3_^2−^ as compared to Cl^−^ and PO_4_^3−^ ions. The pseudo-first-order model explained better the adsorption of both phosphate and nitrate onto the biochar–MgAl–LDH with a faster removal rate probably with equilibrium attainable quickly, especially at lower initial phosphate–nitrate concentration. The maximum monolayer adsorption capacities of phosphate and nitrate by the non-linear Langmuir model were 177.97 mg/g and 28.06 mg/g at 308 and 298 K, respectively. The regeneration test confirmed that the biochar–MgAl composite can be restored effortlessly and has the potential to be recycled after several treatment sequences. The biochar–MgAl–LDH demonstrated capabilities for higher removal of phosphate and nitrate from real wastewater.

## Figures and Tables

**Table 1:** Composition of biochar–MgAl composites.

Sample Name	Biochar (g)	Mg:Al Salts (g) in 100 mL
B_5_MgAl	5	7.69:3.75
B_10_MgAl	10	7.69:3.75

**Table 2:** Textural characteristics of biochar–MgAl composites.

Parameter	B_5_MgAl	B_10_MgAl
BET surface area (m^2^/g)	441.06	381.13
Pore volume (cm^3^/g)	0.299	0.209
Pore radius (based on Barrett, Joyner, and Halenda (BJH) (nm)	1.55	1.55

**Table 3:** Parameters of kinetic models for phosphate and nitrate adsorption onto B_5_MgAl composite.

Pollutant	*C_o_*		Pseudo First Order ln(q_e_ − q_t_) = ln q_e_ − k_1_t			$\mathrm{Pseudo}\;\mathrm{Sec}\mathrm{ond}\;\mathrm{Order}\;\frac{t}{q_{t}}=\frac{t}{q_{e}}+\frac{1}{k_{2}q_{e}^{2}}$		
	(mg/L)	*q_e_*_(exp)_	*q_e_*_(model)_	*k*_1_	*R* ^2^	*q_e_*_(model)_	*k*_2_ × 10^−5^	*R* ^2^
Phosphate	10	67.44	73.69	0.01	0.951	97.08	1.96	0.936
	30	132.45	123.13	0.006	0.988	172.41	1.66	0.934
	50	145.36	140.97	0.006	0.964	357.14	0.19	0.804
Nitrate	10	31.76	28.26	0.005	0.969	44.05	4.88	0.802
	30	41.12	34.12	0.006	0.960	54.64	3.96	0.916
	50	64.88	56.59	0.007	0.980	87.71	2.58	0.829

**Table 4:** Parameters of linear Langmuir and Freundlich isotherm models for phosphate and nitrate adsorption onto B_5_MgAl composite.

Pollutant	*T* (K)	$\mathrm{Langmuir}\;\frac{C_{e}}{q_{e}}=\frac{q_{m}}{K_{l}}+\frac{C_{e}}{q_{m}}$			$\mathrm{Freundlich}\;lnq_{e}=lnK_{F}+\frac{1}{n}lnC_{e}$		
		*q_max_* (mg/g)	*K_L_*	*R* ^2^	*K_F_*	1/*n*	*R* ^2^
Phosphate	298	146.41	0.32	0.982	59.14	0.22	0.943
	308	177.93	0.46	0.998	70.10	0.28	0.936
	318	180.50	0.59	0.999	77.47	0.26	0.930
Nitrate	298	31.94	0.07	0.960	5.16	0.425	0.980
	308	21.44	0.19	0.976	6.88	0.287	0.911
	318	18.62	0.25	0.999	9.29	0.183	0.890

**Table 5:** Parameters of non-linear Langmuir and Freundlich isotherm models for phosphate and nitrate adsorption onto B_5_MgAl composite.

Pollutant	*T* (K)	$\mathrm{Langmuir}\;q_{e}=\frac{q_{max}\;b\;C_{e}\;}{1+b\;C_{e}}$			$\mathrm{Freundlich}\;q_{e}=K_{F}\;C_{e}^{1/n}$		
		*q*_max_ (mg/g)	*K_L_*	*R* ^2^	*K_F_*	1/*n*	*R* ^2^
Phosphate	298	141.75	0.74	0.972	58.88	0.22	0.980
	308	176.09	0.49	0.999	69.24	0.28	0.968
	318	177.97	0.64	0.999	76.74	0.26	0.965
Nitrate	298	28.06	0.10	0.961	5.14	0.42	0.999
	308	20.78	0.21	0.966	6.98	0.27	0.975
	318	19.00	0.38	0.998	9.30	0.18	0.986

**Table 6:** Thermodynamic parameters for phosphate and nitrate adsorption onto B_5_MgAl composite.

	*T* (K)	*K_d_*	∆*G* (kJ/mol)	∆*H* (kJ/mol)	∆*S* (J/mol K)
Phosphate	298	8.43	−5.28		
	308	9.95	−5.88	17.18	75.23
	318	13.06	−6.79		
Nitrate	298	1.18	−0.42		
	308	1.06	−0.15	−6.73	−21.20
	318	1.006	−0.02		

## Supplementary Materials

The following are available online at https://www.mdpi.com/2079-4991/10/2/336/s1, Figure S1: Effect of co-existing ions 0.01 M (a) and 0.1 M(b) on the percentage removal of nitrate and phosphate onto biochar/MgAl composite.
