# Supplementary material for: Comparative Adsorptive Removal of Phosphate and Nitrate from Wastewater Using Biochar-MgAl LDH Nanocomposites: Coexisting Anions Effect and Mechanistic Studies
Source: Nanomaterials (Basel). 2020 Feb 16;10(2):336. doi: 10.3390/nano10020336 (PMC7075123; doi:10.3390/nano10020336)
Supplement: Supplementary file 1 [file nanomaterials-10-00336-s001.pdf]

Comparative adsorptive removal of phosphate and nitrate from wastewater using biochar-MgAl LDH nanocomposites: coexisting anions effect and mechanistic studies

Omar Alagha[1,*], Muhammad Saood Manzar [1], Mukarram Zubair[1], Ismail Anil[1], Nuhu Dalhat Mu'azu[1], Aleem Qureshi[1]

[1] Environmental Engineering Department, College of Engineering A13, Imam Abdulrahman Bin Faisal University, Main Campus, P.O. Box 1982, Dammam 34212, Saudi Arabia; msmanzar@iau.edu.sa (M.S.M); mzzubair@iau.edu.sa (M.Z); ianil@iau.edu.sa (I.A); nadalhat@iau.edu.sa (N.D.M); aqureshi@iau.edu.sa(A.Q)

* Correspondence: oaga@iau.edu.sa; Tel.: +966506616532

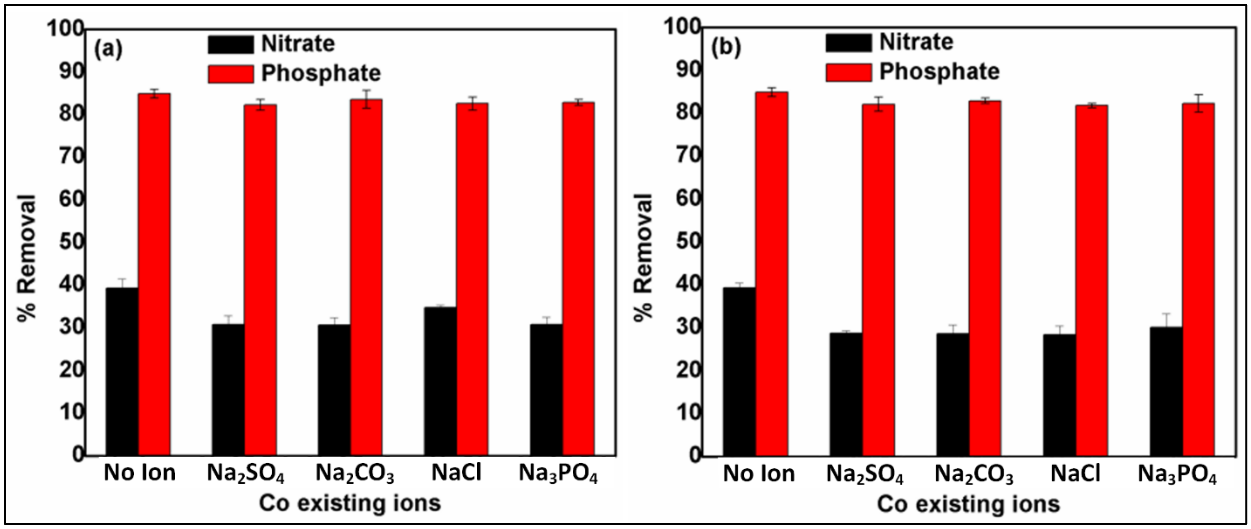

Figure S1. Effect of co-existing ions 0.01 M (a) and 0.1 M(b) on the percentage removal of nitrate and phosphate onto biochar/MgAl composite
